# Targeting intolerance of uncertainty in young children diagnosed with autism: A randomized controlled trial of a parent‐mediated group intervention

**DOI:** 10.1002/jcv2.70027

**Published:** 2025-06-26

**Authors:** Claudia S. Y. Ong, Jacqui Rodgers, Matthew N. Cooper, Zac Dempsey, Rebecca Eaton, Katia Haines, Rebecca Kuzminski, Iliana Magiati, Murray T. Maybery, Mirko Uljarević, John Wray, Andrew J. O. Whitehouse, Gail A. Alvares

**Affiliations:** ^1^ The Kids Research Institute Australia The University of Western Australia Perth Western Australia Australia; ^2^ School of Psychological Science The University of Western Australia Perth Western Australia Australia; ^3^ Population Health Sciences Institute Newcastle University Newcastle UK; ^4^ Department of Psychiatry and Behavioural Sciences Stanford University Stanford California USA; ^5^ WA Department of Health Child Development Service Perth Western Australia Australia

**Keywords:** anxiety, autism spectrum disorders, cognitive therapy, parent‐child relationships, RCT design

## Abstract

**Background:**

Young children diagnosed with autism experience high rates of co‐occurring anxiety, with uncertainty‐related concerns commonly reported. This randomized controlled trial investigated an 8‐week parent‐mediated group anxiety intervention, “Coping with Uncertainty in Everyday Situations” (CUES‐Junior©).

**Methods:**

Parents of 4–7‐year‐old children diagnosed with autism and experiencing uncertainty‐related anxiety were recruited. The primary outcome was change from baseline in blinded assessor ratings of child responses to uncertainty and impact on family, measured post‐intervention and 2‐month follow‐up. Secondary outcomes were parent‐reported child anxiety and intolerance of uncertainty (IU), parental IU and mental health, parenting sense of competence, along with intervention feasibility and acceptability.

**Results:**

Sixty‐four children were randomized to CUES‐Junior© (*n* = 33) or waitlist (*n* = 31); five families withdrew post‐randomization. Immediately post‐intervention, significantly more CUES‐Junior© participants were rated as clinically improved from baseline in child responses to uncertainty (OR = 34.48; 95% CI = 1.72–690.04, *p* = 0.02) and in family impact (OR = 8.99; 95% CI = 1.52–53.05, *p* = 0.02) compared to waitlist. Significant improvements were also observed in parent‐reported child IU and parenting satisfaction, favoring CUES‐Junior©. At subsequent 2‐month follow‐up, CUES‐Junior© participants showed sustained improvements in the impact of uncertainty on children, and parental ratings of child IU and anxiety, parenting sense of competence, and parental stress, compared to baseline. The program was feasible to administer and acceptable to parents.

**Conclusions:**

CUES‐Junior© had an immediate treatment effect on child responses to uncertain situations and impact on families, with maintained improvements observed at follow‐up. This novel mechanism‐targeted and autism‐informed program holds promise for addressing early uncertainty‐related anxiety in young children diagnosed with autism.

## Graphical abstract

In this clinical trial, parents of young children between four and 7 years of age, diagnosed with autism and experiencing anxiety related to uncertainty, completed an 8 week group program. Post‐group, parents reported significant reductions in the impact of uncertainty‐related anxiety on their child and family, with effects also noted on parenting satisfaction, compared to a waitlist control. At follow‐up, significant effects were observed across most parent‐reported child and parent outcomes, suggestive of a potential sleeper effect.

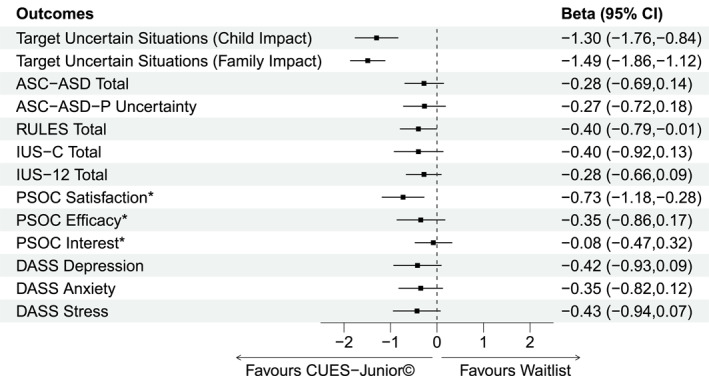

**Key points**

- Intolerance of uncertainty is a key transdiagnostic mechanism contributing to development and maintenance of anxiety, particularly for autistic individuals.
- Children diagnosed with autism commonly experience clinically significant anxiety, however limited early supports are available.
- This randomized controlled trial evaluated a novel parent‐mediated group program targeting intolerance of uncertainty in young children diagnosed with autism.
- Compared to a waitlist condition, children receiving the program were rated as more likely to have clinically improved from baseline post‐intervention in the impact of uncertainty on children and families.
- Findings support the feasibility of intervention targeting uncertainty‐related anxiety in this early developmental period.

## INTRODUCTION

Anxiety frequently co‐occurs in autism^1^ (Lai et al., 2019), a neurodevelopmental condition characterized by differences and difficulties with social communication and interaction, repetitive behaviors, highly focused interests, and/or sensory sensitivities (American Psychological Association, 2022). Elevated and impairing anxiety appears early in life for autistic individuals, with up to two‐thirds of young children diagnosed with autism experiencing clinically significant anxiety (Vasa et al., 2020). Anxiety symptoms are persistent for autistic young people, often increasing in severity over time (Uljarević et al., 2020), and exerting negative functional consequences on participation in everyday activities, academic performance, social outcomes, and family functioning (Adams & Emerson, 2021; Emerson & Adams, 2023).

Several transdiagnostic mechanisms have been proposed to explain the high co‐occurrence of anxiety and autism, including emotion regulation difficulties (Cai et al., 2018), sensory processing differences (Neil et al., 2016), and intolerance of uncertainty (IU; Boulter et al., 2014; Jenkinson et al., 2020). IU refers to a “tendency to react negatively in emotional, cognitive and/or behavioral ways to uncertain situations and events” (p. 216; Buhr & Dugas, 2009), linked to the development, maintenance, and severity of anxiety in the general population (Rosser, 2018), and a leading candidate mechanism underpinning anxiety in autistic people (Jenkinson et al., 2020). In cross‐sectional studies with autistic adolescents and adults, elevated IU is associated with autistic traits (Neil et al., 2016), emotional dysregulation (Cai et al., 2018), and need for predictability and sameness (Wigham et al., 2015). Informant ratings of IU are also higher in school‐aged children on the autism spectrum compared to non‐autistic children (Jenkinson et al., 2020). More recently, uncertainty‐related anxiety has been described in younger children diagnosed with autism (Keefer, Singh, et al., 2024; Vasa et al., 2023), manifesting in more emotional and behavioral responses, and associated with significant child and family functional difficulties (Ong et al., 2023).

While evidence strongly supports cognitive behavioral therapy (CBT) for addressing anxiety concerns in non‐autistic youth (Sigurvinsdottir et al., 2020), modified CBT incorporating autism‐specific adaptations (e.g., visual supports, social coaching, and individualized reinforcement strategies) exhibits more moderate efficacy, considerable treatment response heterogeneity, and variable recovery rates from anxiety disorders (Perihan et al., 2020; Sharma et al., 2021; Warwick et al., 2017). Of note, larger effect sizes have been reported for CBT programs for autistic youths that incorporate a higher degree of parental/family involvement compared to child‐only programs (Perihan et al., 2020). One potential explanation for such substantial variability in treatment response rates may be in targeting broader anxiety symptoms rather than the specific underlying mechanisms contributing to anxiety, such as IU. Supporting this, a previous study observed that higher IU pre‐intervention predicted poorer outcomes from modified CBT for anxiety in autistic youth (Keefer et al., 2017). IU‐focused interventions, therefore, represent an opportunity to directly reduce anxiety in autistic individuals.

Originally designed for school‐aged children and adolescents diagnosed with autism, the Coping with Uncertainty in Everyday Situations (CUES©) parent‐mediated group program was developed as a targeted mechanism‐informed anxiety intervention (Rodgers et al., 2017). Through parental input and support, CUES© aims to increase an autistic young person's capacity to successfully cope with uncertain situations causing anxiety or distress. A recent CUES© feasibility trial (*n* = 50) reported significant improvements in blinded assessor ratings of children's responses to uncertainty at 26‐week follow‐up, with moderate effect sizes, compared to a comparison group receiving enhanced care as usual (Rodgers et al., 2023). Whilst feasibility, acceptability, and preliminary efficacy has been reported, the potential downward extension of CUES© to younger children has not yet been evaluated.

Indeed, despite increasing evidence highlighting the emergence of anxiety in young children diagnosed with autism, and the potential for interventions in this developmental period to alter longer‐term mental health trajectories (Howes Vallis et al., 2020), few interventions have been evaluated for this age group (Vasa et al., 2020). Growing evidence supports modified CBT targeting anxiety for younger children on the autism spectrum (Cook et al., 2019; Driscoll et al., 2020; Simpson et al., 2023), with promising feasibility and preliminary efficacy recently reported in programs incorporating IU‐focused content (Adams et al., 2024; Charman et al., 2021; Keefer, Perrin, et al., 2024; Simpson et al., 2023). Two studies, DINOSAUR (Keefer, Perrin, et al., 2024) and Predictive Parenting (Charman et al., 2021), investigated novel autism‐informed multicomponent programs, with both incorporating IU‐focused therapeutic processes, whilst two studies evaluated feasibility (Simpson et al., 2023) and preliminary efficacy (Adams et al., 2024) of an adapted Cool Little Kids program (CLK‐CUES), which incorporated additional sessions focusing on strategies to cope with uncertainty. For these latter studies (Adams et al., 2024; Simpson et al., 2023), a notable feature was the lack of exclusion criteria for children, with children not required to meet a diagnostic threshold for anxiety for parents to participate in the program. Whilst these previous and very recent studies demonstrate the feasibility of delivering IU‐focused content to younger children, no previous study has specifically, and directly, targeted uncertainty‐related anxiety. Thus, the extent to which IU may represent a viable therapeutic target to reduce anxiety in young children diagnosed with autism remains unknown.

The present study aimed to investigate the efficacy of a developmentally adapted version of CUES© (“CUES‐Junior©”) for young children aged 4–7 years diagnosed with autism. We hypothesized that: (i) children randomized to receive the CUES‐Junior© program would demonstrate greater improvements in blinded assessor‐ratings of the impact of uncertainty related anxiety on children and families, compared to a waitlist condition, post‐intervention (primary outcome); (ii) CUES‐Junior© would reduce parental ratings of IU and anxiety in children and parents, and improve parental self‐efficacy and mental health, post‐intervention (secondary outcomes); and (iii) observed gains from baseline in the CUES‐Junior© group would be maintained at follow‐up.

## METHODS

### Trial design

**FIGURE 1:**
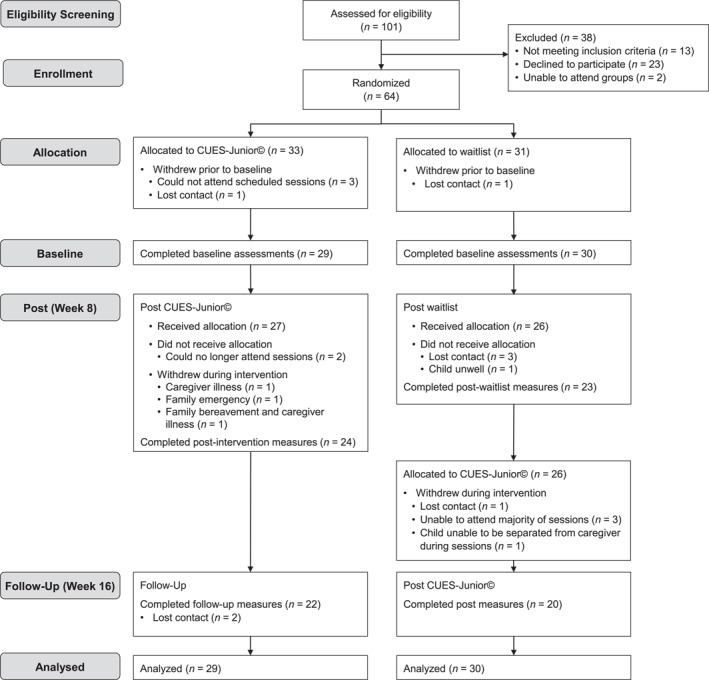
CONSORT participant recruitment and flowchart.

The trial was registered on the Australian New Zealand Clinical Trials Registry (ANZCTR; ACTRN12621000294853). Ethics was obtained from the Child and Adolescent Health Service Human Research Ethics Committee (RGS0000004228) in Western Australia. Parents/primary caregivers (“parents”) provided written informed consent. This study was a parallel two‐group single‐site trial comparing the CUES‐Junior© group program to an 8‐week waitlist. Families randomized to waitlist were offered a place in the next available CUES‐Junior© group program after their 8‐week waiting period. Group sessions were delivered through CliniKids (a community clinical service for children on the autism spectrum or with developmental delays), at The Kids Research Institute Australia in Perth, Western Australia. Measures were collected at baseline (post‐randomization), post‐intervention/post‐waitlist (8 weeks post‐baseline), and follow‐up (16 weeks post‐baseline; intervention group only); see Figure 1 CONSORT flow diagram.

### Participants

Inclusion criteria were parents of children diagnosed with autism where: (i) child was aged 4–7 years at consent, (ii) child experienced anxiety, worries, fears, or distress relating to uncertainty; and (iii) parents had adequate spoken and written English to access the intervention. Eligibility was assessed by researcher (GA, CO) screening of parent‐reported child difficulties with uncertainty‐related anxiety, causing functional impact(s) for the child and/or family. Exclusion criteria, based on factors likely to limit participation in program activities, were: (i) children with intellectual disability or global developmental delay requiring significant functional supports; (ii) parent experiencing severe or complex medical, health, or mental health issues; (iii) family not intending to remain in Perth for the study duration; and (iv) family involved in active court proceedings or special care arrangements.

### Procedure

#### Sample size

Sample size estimation, and calculation inputs, were informed based a prior CUES© feasibility study (Rodgers et al., 2017) and meta‐analyses of anxiety interventions for autistic youth (Perihan et al., 2020; Ung et al., 2015). A sample size of 60 was required to have 80% power to detect an effect of 0.75 with a two‐group design and significance of 0.05.

#### Recruitment

Families were recruited between July 2021 and March 2023 via flyers and emails through CliniKids, social media advertising, and snowball recruitment with participants of previous research. After telephone pre‐screening to assess eligibility, informed consent was obtained electronically using the e‐consent framework on REDCap (Research Electronic Data Capture; Harris et al., 2009).

#### Randomization

After obtaining informed consent, participants were randomized to either CUES‐Junior© or waitlist by GA using a computer‐generated four‐block randomization schedule using Sealed Envelope and the randomization module within REDCap.

#### Group allocation and blinding

Groups were scheduled based on therapist and family availabilities. Ten groups were scheduled across October 2021 to May 2023, with last participant follow‐up in July 2023. One group was discontinued due to high participant attrition, occurring alongside a surge of COVID‐19 cases within Western Australia across mid‐2022. Across the intervention period, changes in social distancing, masks indoors, and maximum room capacity fluctuated.

Families were not blinded to randomization. The four assessors rating the primary outcome were blinded to both group allocation and timepoint, and therapists delivering the intervention were unaware of allocation. Statisticians (MC, ZD) conducting data analyses were independent of the team administering the intervention and collecting data.

### Intervention

CUES‐Junior©, a developmental adaptation of CUES©, is a parent‐mediated manualised group program aiming to increase children's capacity to cope with everyday uncertainty and develop increased flexibility to uncertainty across a range of settings (Rodgers et al., 2017). CUES© supports parental understanding and awareness of the nature and impact of autism‐related anxiety relating to IU, and utilization of strategies aimed at gradually increasing children's ability to cope with uncertainty. Adaptation involved a rigorous process of integrating input from stakeholder consultations (parents and clinicians), parent focus groups (Ong et al., 2023), and a feasibility study (*unpublished*). Key adaptations included simplification of strategies (e.g., using more concrete representations), greater emphasis on behavioral rather than cognitive strategies, and increased use of developmentally appropriate content (e.g., attachment, parental co‐regulation, emotion coaching, and promoting positive parent‐child interactions).

CUES‐Junior© consisted of eight two‐hour weekly sessions delivered to small groups, between five to eight families. Each session involved a review of home tasks from the previous week (from week two), a mixture of presentation and collaborative group discussion of the session's content with accompanying handouts and tools (e.g., a weekly monitoring sheet, visual supports, story scripts, and play scripts), individualization of strategies, group coaching, and mindfulness activities. Parents who missed sessions were offered a call with a facilitator to review missed content. Each group was facilitated by two therapists, with at least one being a registered clinical psychologist. The facilitator team included four clinical psychologists and six trainee clinical psychologists, all with considerable autism knowledge and experience. All facilitators had received training in the original CUES© program and the adapted manual. Trainee psychologists were supervised regularly by registered clinical psychologist supervisors.

Groups were delivered independently of the original CUES© developer (JR) and the researchers collecting outcome measures. All sessions were video recorded, with 10% of sessions randomly selected across therapist, session, and group for fidelity evaluation (assessed by JR at trial end). A pre‐defined fidelity checklist (Rodgers et al., 2023), which all therapists were blind to, assessed adherence to manual content (0 = not at all, 1 = briefly or insufficiently covered, 2 = covered adequately) and group therapeutic processes (0 = not at all; 1 = minimal evidence, 2 = several examples).

### Measures

Questionnaires were completed electronically on REDCap, with clinical interviews for the primary outcome conducted over the telephone, lasting up to 60 min. Measures of child and family characteristics were collected at baseline, with primary and secondary outcomes collected at baseline, post, and follow‐up timepoints.

#### Primary outcome

Derived from semi‐structured clinical interviews conducted at three timepoints (baseline, post, follow‐up), the primary outcome was assessor ratings of change from baseline in (i) child responses to uncertain situations and (ii) the impact of child responses to uncertainty on families. Interview questions were identical to prior CUES© studies (Rodgers et al., 2017, 2023), with the coding protocol developed by the Research Unit on Pediatric Psychopharmacology and Psychosocial Interventions (Arnold et al., 2003). Interviews (conducted by GA or CO) asked parents to identify at least two real‐life situations that were meaningful to them where their child experienced difficulties with uncertainty (“Target Uncertain Situations”). Parents reported on their child's reactions to these situations and subsequent impact on daily functioning and/or social and community participation (Child Impact), along with the impact on parent or family functioning (Family Impact). See Table S4 for brief examples of situations reported and impact of these. Responses were then summarized into vignettes, with researchers (GA, CO) reviewing each vignette to ensure no information was included that could potentially reveal group allocation or timepoint. At the end of the trial, assessors (three research staff and a doctoral‐level student, all with at least undergraduate training in allied health disciplines and experience with autism research or disability support work) were provided with vignette pairs (one from baseline and either post‐intervention/post‐waitlist or follow‐up). Assessors were initially trained by author JR on how to rate vignettes, with each assessor coding a range of example vignettes to achieve consensus coding. After training, each assessor independently read each vignette pair and determined change from baseline for Child Impact and Family Impact using a 9‐point scale (Arnold et al., 2003) (1 = very much improved, 2 = markedly improved, 3 = definitely improved, 4 = equivocally improved, 5 = no change, 6 = equivocally worse, 7 = definitely worse, 8 = markedly worse, 9 = disastrously worse); scores were then averaged across the four assessors. Intraclass correlation coefficient estimates for estimating interrater reliability were excellent across timepoint and child/family rating (ranging between 0.94 and 0.95). Averaged scores were classified into “improved” (scores 1–3), “little/no change” (scores 3.01–6.99), and “worse from baseline” (scores 7–9). No participants were rated as “worse from baseline” at either timepoints, so ratings were subsequently classified as “improved” (“responder”) and “little/no change” (“non‐responder”).

#### Secondary outcomes

Parent‐reported child outcomes included the 17‐item Responses to Uncertainty and Low Environmental Structure (RULES; Sanchez et al., 2017), an IU measure in young children (score range 17–85; higher scores = higher IU); the 27‐item Intolerance of Uncertainty Scale–Children (IUS‐C; Comer et al., 2009), measuring children's IU and tendency to react negatively emotionally, cognitively, and behaviorally to uncertain situations (score range 27–135; higher scores = higher IU); and the 24‐item Anxiety Scale for Children–ASD Parent version (ASC‐ASD‐P; Rodgers et al., 2016), with four subscales: Separation Anxiety, Uncertainty, Performance Anxiety, and Anxious Arousal (total scores range 0–72; ≥20 = significant anxiety, >24 = clinical anxiety; uncertainty subscale score range 0–24).

Self‐reported parent outcomes included the 17‐item Parenting Sense of Competence (PSOC; Johnson & Mash, 1989), containing three subscales: Satisfaction, Efficacy, and Interest (higher score = higher parenting satisfaction/self‐efficacy; score range 9–54, satisfaction; 8–48, efficacy; 2–12, interest); the 21‐item Depression Anxiety Stress Scale (DASS‐21; Lovibond & Lovibond, 1995), with Depression, Anxiety and Stress subscales (subscale score range 0–21; higher scores = greater symptoms); and the 12‐item Intolerance of Uncertainty Scale‐12 (IUS‐12; Carleton et al., 2007), measuring parental IU (score range 12–60; ≥35 = clinically significant IU).

Where there was no protocol from a relevant publication or manual, individual item missing values were imputed with individual mean item subscale scores.

#### Child and family characteristics

Demographics were collected at baseline, along with the Social Responsiveness Scale (SRS‐2; Constantino & Gruber, 2012), Repetitive Behavior Questionnaire‐2 (RBQ‐2; Leekam et al., 2007), and Vineland Adaptive Behavior Scales 3^rd^ edition parent‐caregiver form (VABS‐3; Sparrow et al., 2016), measuring social‐communication and behavioral autism‐related traits and level of functioning, respectively.

#### Feasibility and acceptability

Feasibility was assessed through participant retention and attendance. Acceptability was assessed through weekly satisfaction and feedback questionnaires.

### Statistical analyses

Intention‐to‐treat (ITT) analyses followed a pre‐specified analysis plan, undertaken using R version 4.3.1, conducted by clinical trial statisticians (MC, ZC). Minor differences between the analysis plan described in the clinical trial registration and the analyses conducted on the outcomes below were discussed with the statisticians and decided on prior to any analyses being conducted. Throughout, effect size estimates are reported with 95% confidence intervals (CIs).

Group differences in the primary outcome (post‐intervention) were analyzed using logistic regression controlling for a priori identified covariates (child's age, gender, SRS‐2 raw total) with participants classified as improved from baseline/“responder” or little‐to‐no change from baseline/”non responder.” Maximum Penalized Likelihood Estimation using Jeffery's invariant prior was used to better handle low cell counts present in the data (Kosmidis & Firth, 2021). Group differences in the secondary (continuous) outcomes were analyzed using ANCOVA, with the post‐intervention value being the outcome variable in a linear regression model, controlling for the baseline value of the outcome variable and the covariates listed above.

Missing data due to drop‐out were handled using Multivariate Imputation by Chained Equations (50 imputations) with all reported results the pooled effect estimates, unless otherwise stated (Van Buuren & Groothuis‐Oudshoorn, 2011). Sensitivity analyses to evaluate the robustness of results included examining the impact of excluding covariates, adjusting the number of imputations used, and comparing outcomes to complete case analysis. A forest plot figure was used to visually summarize the findings across primary and secondary outcome analyses; to generate this, the continuous scale score (averaged across four assessor blinded ratings) was used for the primary outcome and standardized effects were presented from the same models as detailed above following variable standardization implemented with the datawizard package (Patil et al., 2022). Baseline group differences in parent self‐reported IU‐12 and DASS‐21 subscale scores were also evaluated in sensitivity analyses, with no significant differences in interpretation of findings after adjusting for these additional covariates.

Maintenance of intervention effects (defined as improvement from baseline through follow‐up) was assessed in the CUES‐Junior© group only. For the primary outcome, changes in the proportion of “responders” and “non‐responders” at post‐intervention and follow‐up were examined using McNemar's test, as the small sample size and low cell counts lead to instability in more complex models. For secondary outcomes, linear mixed‐effects models were used to evaluate change from baseline to post‐intervention and follow‐up, using the lme4 package with a random intercept term included in the model (Douglas et al., 2015).

## RESULTS

### Participants

Sixty‐four parents were randomized to CUES‐Junior© (*n* = 33) or waitlist (*n* = 31). Five participants withdrew post‐randomization, prior to completing baseline measures; Figure 1. Groups did not differ significantly on demographic characteristics at baseline apart from primary caregiver status, with more mothers in the waitlist (*p* = 0.02); Table 1. There was a tendency for greater child psychotropic medication use in the CUES‐Junior© group (*p* = 0.05).

**TABLE 1:** Participant demographic and clinical characteristics.

	CUES‐junior© (*n* = 29)	Waitlist (*n* = 30)
Child characteristics		
Age (years)	6.32 (1.06)	5.93 (1.20)
Sex		
Female	7 (24%)	7 (23%)
Male	22 (76%)	23 (77%)
Primary language		
English	27 (93%)	29 (97%)
Others	2 (6.8%)	1 (3.3%)
Age at autism diagnosis (years)	4.70 (1.52)	4.95 (1.55)
Other co‐occurring diagnoses		
ADHD	8 (28%)	12 (40%)
Speech or language delay	9 (31%)	10 (33%)
Global developmental delay	6 (21%)	3 (10%)
Developmental coordination disorder	2 (6.9%)	1 (3.3%)
Psychotropic medication use	17 (59%)	10 (33%)
SRS‐2, total raw score	111.34 (27.07)	108.00 (21.25)
RBQ‐2, total raw score	41.72 (6.18)	42.70 (8.12)
VABS‐3, standard score	73.88 (12.46)	77.4 (7.03)
Family characteristics		
Primary caregiver		
Mother	24 (83%)	30 (100%)
Father	3 (10%)	0 (0%)
Other	2 (6.4%)	0 (0%)
Age (years)	38.61 (9.41)	40.23 (6.17)
Highest level of education		
Secondary	2 (7.1%)	4 (13%)
Vocational	4 (14%)	7 (23%)
University	22 (79%)	19 (63%)
Employment		
Full‐time	2 (6.9%)	2 (6.7%)
Part‐time	15 (52%)	11 (37%)
Not currently working (leave/unemployed)	12 (41%)	17 (57%)
Family cultural and ethnic identity^a^		
White Australian	18 (62%)	20 (67%)
Australian Aboriginal	1 (3.4%)	1 (3.3%)
North‐East Asian	2 (6.9%)	2 (6.7%)
North‐West European	3 (10%)	4 (13%)
South‐East Asian	3 (10%)	0 (0%)
Other	2 (6.9%)	3 (10%)
Parent diagnoses		
Autism	0 (0%)	5 (17%)
ADHD	4 (14%)	8 (27%)
Mood and/or anxiety disorder(s)	10 (34%)	17 (57%)
Socioeconomic status^b^	24 (96%)	24 (89%)

*Note*: Values are *n* (%) or mean (SD).

Abbreviations: ADHD, attention deficit hyperactivity disorder; RBQ‐2, repetitive behavior questionnaire‐2; SRS‐2, social responsiveness scale; VABS‐3, vineland adaptive behavior scales 3rd edition.

^a^ Australian Standard Classification of Cultural and Ethnic Groups, Australian Bureau of Statistics.

^b^ Socio‐economic Indexes for Areas (SEIFA), % of participants classed as not socially disadvantaged based on postcode on the Index of Relative Socioeconomic Disadvantage (Australian Bureau of Statistics, 2021).

#### Primary outcome

Adjusting for autism severity, age, and gender, eight (33%) children in the CUES‐Junior© group showed significant clinical improvement in blinded assessor‐rated child responses to uncertainty post‐intervention, compared to 0% in the waitlist group (OR = 34.48; 95% CI = 1.72, 690.04, *p* = 0.02); Table 2. Nine (38%) CUES‐Junior© participants showed clinical improvement in the assessor‐rated family impact of child uncertainty responses, compared to one (5%) in waitlist (OR = 8.99; 95% CI = 1.52, 53.05, *p* = 0.02); Table 2. No participants were rated as declined or worse than baseline on the primary outcome following the intervention or waitlist period.

**TABLE 2:** Proportion of assessor‐rated “improved” (“responder”) and “no change” (“non‐responder”), relative to baseline, on the primary outcome at post‐intervention and follow‐up.

Post‐intervention							
	CUES‐junior© (*n* = 24)		Waitlist (*n* = 20)		Between group comparison^a^		
	Improved	No change	Improved	No change	OR	95% CI	*p*
Child impact	8 (33%)	16 (67%)	0 (0%)	20 (100%)	34.48	1.72, 690.04	0.02
Family impact	9 (38%)	15 (63%)	1 (5%)	19 (95%)	8.99	1.52, 53.05	0.02

Across baseline to follow‐up (CUES‐Junior© group only; *n* = 22)						
	Post‐intervention		Follow‐up		Within group comparison^b^	
	Improved	No change	Improved	No change	*X* ^2^ (1, *N* = 22)	*p*
Child impact	7 (32%)	15 (68%)	13 (59%)	9 (41%)	4.17	0.04
Family impact	8 (36%)	14 (64%)	13 (59%)	9 (41%)	1.45	0.22

^a^ Pooled estimate from logistic regression model with imputation (*m* = 50) for missing data.

^b^ McNemar's test on complete case data for those assessed at all three timepoints.

At follow‐up for the CUES‐Junior© participants, all seven (100%) children who were rated as clinically improved post‐intervention on the assessor‐rated child responses to uncertainty sustained their “improved” rating status at follow‐up and a further six (40%) who had a “no change” status at post‐intervention showed improvement at follow‐up (59% of all participants in the CUES‐Junior© group; *Χ* ^2^ (1, *N* = 22) = 4.17, *p* = 0.04); Table S1 and Table 2. With the assessor‐rated family impact of child's uncertainty responses, five (63%) children who were rated as clinically improved post‐intervention showed sustained improvements at follow‐up, with three (38%) reduced to a “no change” status. Further, eight of 14 (57%) who had a “no change” status post‐intervention showed improvement at follow‐up, while six (43%) CUES‐Junior © participants continued to demonstrate “no change” at follow‐up (*Χ* ^2^ (1, *N* = 22) = 1.45, *p* = 0.23); Table S1 and Table 2.

#### Secondary outcomes

**FIGURE 2:**
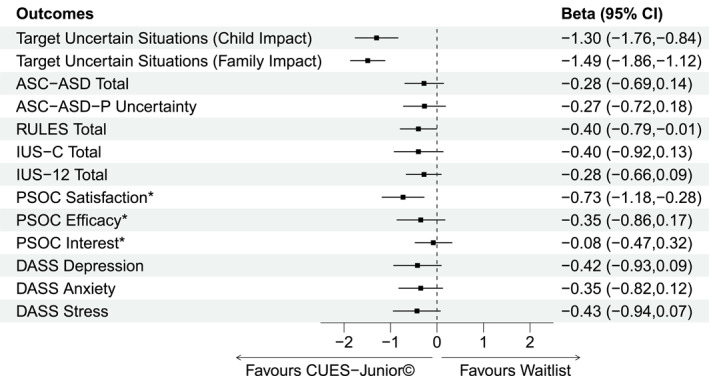
Effect sizes for primary and secondary outcomes post‐intervention. Standardized effect sizes are beta coefficients (95% confidence intervals) from a linear regression (ANCOVA framework) where all variables in the model (independent and dependent) are scaled (standardized to 0, 1) prior to the model being run. For the primary outcome, the underlying (average) continuous scale score was used for the primary outcome (Target Uncertain Situations, Child and Family Impact). ASC‐ASD‐P, anxiety scale for children–ASD parent version; DASS, depression anxiety stress scale; IUS‐12, intolerance of uncertainty scale‐12 item; IUS‐C, intolerance of uncertainty scale–children; PSOC, parenting sense of competence; RULES, responses to uncertainty and low environmental structure. *PSOC scores have been reversed for comparison across outcomes.

Post‐intervention, significant improvements favoring CUES‐Junior© were observed in parent‐reported child IU symptomatology (RULES; −5.14, 95% CI = −10.12, −0.14, *p* = 0.02) and parenting satisfaction (PSOC satisfaction; 4.41, 95% CI = 1.60, 7.22, *p* < 0.001). No other significant group effects on other secondary parental or child measures were observed; these are presented as standardized effects, alongside the underlying (average) continuous scale score for the primary outcome variable, in Figure 2. See Table S2 for secondary outcome descriptives by timepoint.

At follow‐up, for participants in the CUES‐Junior© group, significant sustained or delayed improvements were observed, relative to baseline, across secondary measures of child IU and anxiety (RULES; −10.76, 95% CI = −14.58,−6.95, *p* < 0.01; IUS‐C; −11.93, 95%CI = −20.51,−3.36, *p* < 0.01; ASC‐ASD‐P; −7.47, 95% CI = −11.31, −3.63, *p* < 0.01; ASC‐ASD‐P Uncertainty; −3.45, 95% CI = −4.88, −2.03, *p* < 0.01); and for parental self‐efficacy (1.42, 95% CI = 0.11, 2.73, *p* = 0.03), satisfaction (3.51, 95%CI = 1.34, 5.68, *p* < 0.01), and parental stress (−1.95, 95% CI = −3.35, −0.55, *p* < 0.01), associated with moderate to large effect sizes; Table 3.

**TABLE 3:** Secondary child and parent outcomes (a) from baseline to post‐intervention and (b) across baseline to follow‐up.

	(a) Post‐intervention CUES‐Junior© versus waitlist				(b) Across baseline to follow‐up within CUES‐Junior© group							
	Group effects				Post‐intervention				Follow‐up			
	Estimate	95% CI	*B*	*p*	Estimate	95% CI	*B*	*p*	Estimate	95% CI	*B*	*p*
Child outcomes												
RULES	−5.14	−10.12, −0.14	−0.40	0.04	−7.05	−10.62, −3.48	−0.55	<0.01	−10.76	−14.58, −6.95	−0.84	<0.01
IUS‐C	−8.10	−18.98, 2.77	−0.40	0.14	−5.62	−14.40, 3.13	−0.29	0.20	−11.93	−20.51, −3.36	−0.62	<0.01
ASC‐ASD‐P total	−3.30	−8.25, 1.65	−0.28	0.18	−3.80	−7.54, −0.08	−0.35	0.05	−7.47	−11.31, −3.63	−0.68	<0.01
ASC‐ASD‐P uncertainty	−1.40	−3.71, 0.92	−0.27	0.22	−1.52	−3.05, 0.01	−0.29	0.05	−3.45	−4.88, −2.03	−0.66	<0.01
Parent outcomes												
PSOC self‐efficacy	1.29	−0.62, 3.20	0.35	0.18	2.18	0.81, 3.56	0.56	<0.01	1.42	0.11, 2.73	0.37	0.03
PSOC satisfaction	4.41	1.60, 7.22	0.73	<0.01	2.56	0.32, 4.81	0.48	0.03	3.51	1.34, 5.68	0.65	<0.01
PSOC interest	0.20	−0.84, 1.24	0.08	0.70	−0.17	−1.00, 0.66	−0.07	0.69	−0.31	−1.17, 0.56	−0.13	0.48
DASS depression	−1.67	−3.71, 0.37	−0.42	0.10	−0.23	−1.60, 1.15	−0.07	0.74	−0.01	−1.46, 1.45	−0.01	0.98
DASS anxiety	−1.41	−3.35, 0.52	−0.35	0.14	−0.39	−1.33, 0.55	−0.14	0.41	−0.11	−1.04, 0.84	−0.04	0.82
DASS stress	−2.18	−4.73, 0.36	−0.43	0.09	−1.29	−2.72, 0.13	−0.32	0.08	−1.95	−3.35, −0.55	−0.49	<0.01
IUS‐12	−3.27	−7.60, 1.05	−0.28	0.13	−0.23	−3.04, 2.58	−0.03	0.87	0.26	−3.12, 3.64	0.04	0.88

Abbreviations: ASC‐ASD‐P, anxiety scale for children‐ASD parent‐report; DASS, depression, anxiety and stress scale 21‐item version; IUS‐12, intolerance of uncertainty scale‐12 item version; IUS‐C, intolerance of uncertainty scale‐children; PSOC, parenting sense of competence; RULES, response to uncertainty and low environmental structure.

#### Feasibility, acceptability, and program fidelity

In terms of intervention adherence, 22 (75.9%) parents in the CUES‐Junior© group completed the 8‐week program, with high attendance (mean number of sessions attended = 6.59 (SD: 1.40)). Of those who missed sessions, 64.5% completed individual sessions to review missed content. Parental satisfaction ratings on weekly content were very high; Table S3.

Therapy fidelity (percentage of items achieving the maximum score on fidelity checklists), was extremely high on adherence to manual content (99.2%) and delivery of therapeutic best practice (99.6%).

## DISCUSSION

The present trial evaluated the efficacy of an autism‐informed, mechanistic intervention targeting child IU and associated distress, delivered to parents of young children diagnosed with autism. Post‐intervention, children who received the CUES‐Junior© program were more likely to be rated by blinded assessors as clinically improved from baseline on their responses to uncertainty and on the associated impact on families compared to waitlist families (primary outcome), with large effect sizes. Significant changes favoring the CUES‐Junior© group were also observed in secondary parent‐reported child IU and parenting satisfaction, with medium and large effect sizes respectively. No other significant group differences were observed post‐intervention. At follow‐up, a greater proportion of children in the CUES‐Junior© group were rated as “improved” on child responses to uncertainty, compared to post‐intervention. Significant improvements from baseline on child IU and anxiety, parenting satisfaction, self‐efficacy, and stress were also reported, associated with moderate to large effects, suggestive of sustained improvements across child and parent outcomes. The program was feasible to deliver in this young population, with high parental satisfaction and therapist fidelity.

There are a few possible explanations for larger intervention effects post‐intervention on the primary outcome, relative to smaller effects on some secondary child and parent outcomes. Firstly, the CUES‐Junior© program targets specific uncertain situations identified by families that are distressing and impactful to the child and family. The degree of impact this anxiety causes, and subsequent changes in this, are more likely to be meaningfully captured using qualitative clinical interviews rather than through questionnaires that assess broader anxiety experiences or features. Secondly, the program's psychoeducational components and weekly monitoring tasks, aimed at increasing parental awareness of IU in children, may have increased parents' precision in more accurately identifying and reporting on IU and anxiety for their child in interviews post‐intervention. Further, IU encompasses affective, behavioral, and cognitive features, which may be expressed differently or variably in younger children, depending on developmental stage, and cognitive or verbal abilities (Ong et al., 2023). There is likely considerable variability in how IU‐related anxiety presents in this young population (Vasa et al., 2020), which may explain variable effects observed across the three quantitative measures of children's IU and anxiety.

Some children in the CUES‐Junior© group were initially classified post‐intervention as “little/no change”/“non‐responders,” but were later rated as “improved”/“responders” at follow‐up; moderate to large improvements on child and parent outcomes relative to baseline were also observed at follow‐up. Together, these findings appear consistent with a “sleeper effect” (van Aar et al., 2017). In the context of parent‐mediated interventions, upskilling parents on strategies to support child development require time, practice, and consolidation (Cook et al., 2019; van Aar et al., 2017). For example, similar sleeper effects were reported in a trial of “Fun with Feelings,” where significant reductions in children's anxiety were observed only at follow‐up (Cook et al., 2019). In the present study, many secondary measures exhibited greater improvement from baseline to follow‐up, than was observed at post‐intervention, for the CUES‐Junior© group. Parent‐mediated interventions necessitate skill transfer from therapists to parents, and subsequently to children, involving a considerable degree of parental modeling, coaching, practice, consistency, and repetition to facilitate generalization; all of which may require time post‐intervention to consolidate and apply in daily life (Chan et al., 2023).

In contrast to the intervention effects on Child Impact at follow‐up, findings on Family Impact were mixed. While some participants who were rated as “improved” on Family Impact post‐intervention maintained this status at follow‐up, some did not; others were rated as “improved” at follow‐up only. This suggests variability in how young children's responses to uncertainty impact family functioning over time. For some, whilst sustained improvements in child's responses to uncertainty were reported, these may not translate to sustained improvements for families, as measured in this study. Caregiving responsibilities, dynamic and cumulative stressors, and family adjustment (Ilias et al., 2018), all may influence how parents perceive their child's IU impacting the family system. While much attention has focused on the benefits of parent‐mediated interventions, the implicit therapy demands placed on parents in implementing such strategies, and differences in each family's capabilities and resources to do so, requires further investigation (Chan et al., 2023; Jurek et al., 2023).

While not designed to evaluate efficacy, a recent feasibility pilot of CUES© for autistic youth (Rodgers et al., 2023) reported no significant between‐group effects immediately post‐intervention, with moderate effects reported at a 26‐week follow‐up. By comparison, the present study observed large between‐group effects on young children's responses to uncertainty and family impact immediately post‐intervention, with effects on child responses maintained at follow‐up for the intervention group. The present findings also complement findings from recently published anxiety interventions incorporating uncertainty‐related content (Adams et al., 2024; Charman et al., 2021; Keefer, Perrin, et al., 2024; Simpson et al., 2023). Together, these trials suggest the value of anxiety supports focused on addressing underlying mechanisms, such as IU, during early sensitive periods of increased developmental malleability, providing opportunities to support parenting practices in ways that may positively influence children's trajectories of wellbeing (Howes Vallis et al., 2020). Strategies within the program, including gradual management of uncertainty, parental co‐regulation, emotion coaching, and graded exposure techniques, hold promise in alleviating some of the distress associated with uncertainty in this population.

In the present study, intervention responder rates at follow‐up (59% in child and family impact) demonstrate similar efficacy to responder rates reported in previous similar clinical trials. For example, 54.4% of young children receiving DINOSAUR, targeting IU and parent accommodation, improved on a global anxiety severity scale (Keefer, Perrin, et al., 2024), and intervention responder rates between 58% and 75% have been reported in meta‐analyses of CBT in autistic youths (Ung et al., 2015). While promising, children rated as showing little or no clinical change by follow‐up raises questions about the factors that impact anxiety intervention efficacy for children diagnosed with autism (Sharma et al., 2021). In addition to possible sleeper effects, developmental factors such as cognitive and language skills, not assessed in this study, can influence intervention outcomes in autistic individuals (Trembath et al., 2019). Parent motivation, parent beliefs and attitudes toward therapy, goodness‐of‐fit between parent and child's needs, and therapeutic alliance between therapists and families, may influence CBT outcomes for school‐aged children diagnosed with autism (Chan et al., 2023). Family capacity and resources, parental motivation, and attitudes toward therapy (Chan et al., 2023; Jurek et al., 2023), all impact effectiveness and engagement, and should be considered in future studies to determine families for whom parent‐mediated programs may be most beneficial.

The present study has several strengths. Firstly, compared to broader or multicomponent anxiety interventions, this autism‐informed, developmentally considered, and theoretically‐driven program targeted IU, a known mechanism contributing to anxiety in autistic people (Boulter et al., 2014). Secondly, while CUES‐Junior© is a manualised group program, therapists adopt an individualized approach, targeting specific situations that are meaningful for families. Thirdly, we included children with co‐occurring developmental or language delays, reflecting the diverse clinical profiles of young children presenting for psychological services. Lastly, the primary outcome combined semi‐structured parent interviews with blinded assessor‐ratings, rather than solely relying on parent‐reported questionnaires; type of informant (parent, child, clinician) is a known factor influencing magnitude of effect sizes in this field (Ung et al., 2015).

Interpretations about the sustained effects of CUES‐Junior© are limited by the lack of follow‐up assessment for the waitlist condition, as findings may be time, rather than intervention, effects. Participants were mostly mothers from more advantaged backgrounds, in caregiving roles or working part‐time, which limits generalizability to families with more limited capacity to participate in parent‐mediated interventions. The reliance on parent‐reported outcomes without direct child observations may also limit conclusions about changes in children's behaviors and internal experiences, although the primary outcome was rated blind to group allocation. Additionally, children were not assessed at baseline to determine whether they reached a clinical threshold for anxiety disorder(s); our inclusion criteria was parent‐reported child anxiety related to uncertainty, causing functional impact for the child and/or family, and it is therefore possible that some children may not have met clinical threshold for an anxiety disorder. However, we adopted a pragmatic and inclusive approach to the inclusion criteria for this study, to better align with how children and families present for services in community healthcare settings, such as the one in which this trial was conducted. Future studies could adopt a multi‐informant approach, including clinician, teacher, and children's reports, where appropriate, and consider assessing how parents utilize learned skills after intervention ceases.

## CONCLUSION

The present study demonstrated that CUES‐Junior© was superior to waitlist in assessor‐rated changes in child responses to uncertainty and family impact immediately post‐intervention. A potential sleeper effect was also observed, with sustained improvements across most child and parent outcomes for the CUES‐Junior© group. These findings advance evidence for mechanism‐informed anxiety supports for young children diagnosed with autism. While group findings overall supported the program's efficacy, next steps will be to investigate predictors of intervention non‐response and moderators of efficacy. Future trials, incorporating larger samples, should also consider designs that incorporate an active comparison group, additional timepoints to evaluate longitudinal and sleeper effects over time, and implementation studies to understand the broader scalability of such early interventions when delivered in community mental health and developmental services.

## AUTHOR CONTRIBUTIONS

**Claudia S.Y. Ong**: Data curation; formal analysis; investigation; project administration; visualization; writing—original draft; writing—review and editing; **Jacqui Rodgers**: Conceptualization; funding acquisition; methodology; resources; supervision; writing—review and editing; **Matthew N. Cooper**: Formal analysis; visualization; writing—review and editing; **Zac Dempsey**: Formal analysis; visualization; writing—review and editing; **Rebecca Eaton**: Investigation; supervision; writing—review and editing; **Katia Haines**: Funding acquisition; supervision; writing—review and editing; **Rebecca Kuzminski**: Conceptualization; funding acquisition; supervision; writing—review and editing; **Iliana Magiati**: Conceptualization; funding acquisition; supervision; writing—review and editing; **Murray T. Maybery**: Supervision; writing—review and editing; **Mirko Uljarević**: Conceptualisation; writing—review and editing; funding acquisition; **John Wray**: Funding acquisition; writing—review and editing; **Andrew J. O. Whitehouse**: Conceptualisation; methodology; writing—review and editing; supervision; resources; funding acquisition; **Gail A. Alvares**: Conceptualization; data curation; formal analysis; funding acquisition; investigation; methodology; project administration; supervision; writing—original draft; writing—review and editing.

## CONFLICT OF INTEREST STATEMENT

The Coping with Uncertainty in Everyday Situations (CUES©) program was co‐developed by Prof. Rodgers; Prof. Rodgers contributed to the original conceptualization of the study and adaptations made to the original program, conducted therapist fidelity, provided clinical supervision, and contributed to analysis interpretation, and was not involved in collection of primary or secondary outcome data, nor in conducting analyses. The funding body of the study had no role in study design, data collection, data analysis, data interpretation, or writing of the manuscript. The remaining authors have reported no biomedical financial interests or potential conflicts of interest.

## ETHICAL CONSIDERATIONS

Ethics was obtained from the Child and Adolescent Health Service Human Research Ethics Committee (RGS0000004228) in Western Australia. Parents/primary caregivers (“parents”) provided written informed consent.

## TRIAL REGISTRATION

The trial was registered on the Australian New Zealand Clinical Trials Registry (ANZCTR; ACTRN12621000294853).

## Supporting information

Supporting Information S1

## Data Availability

The data that support the findings of this study are available from the corresponding author upon reasonable request.

## ENDNOTE

^1^
Autism terminology was informed by language guidelines (Autism Cooperative Research Center; https://www.autismcrc.com.au/) and input from the research team's consumer reference group. For adults, identity‐first language is used (“autistic person”) and for children, “on the autism spectrum,” “with an autism diagnosis” or “diagnosed with autism” is used. “Non‐autistic” refers to both children and adults without an autism diagnosis.
