# Supplementary material for: Targeting intolerance of uncertainty in young children diagnosed with autism: A randomized controlled trial of a parent‐mediated group intervention
Source: JCPP Adv. 2025 Jun 26;6(1):e70027. doi: 10.1002/jcv2.70027 (PMC12973137; doi:10.1002/jcv2.70027)
Supplement: Supplementary file 1 — Supporting Information S1 [file JCV2-6-e70027-s001.docx]

**Supporting Information**

Table S1. Proportion of children classified as ‘Improved’ / ‘No Change’ on the primary outcome post-intervention and follow-up (intervention group only).

| *Child Responses to Uncertainty* | | Follow-Up | |
| --- | --- | --- | --- |
|  |  | Improved | No change |
| Post-Intervention | Improved | 7 (100%) | 0 (0%) |
|  | No change | 6 40(%) | 9 (60%) |
|  | Total | 13 (59%) | 9 (41%) |
| *Family Impact* | | Follow-Up | |
|  |  | Improved | No change |
| Post-Intervention | Improved | 5 (63%) | 3 (38%) |
|  | No change | 8 (57%) | 6 (43%) |
|  | Total | 13 (59%) | 9 (41%) |

Table S2. Descriptives of secondary child and parent outcomes by timepoint.

|  | CUES-Junior© (*n*=29) | | |  | Waitlist (*n*=30) | |  |
| --- | --- | --- | --- | --- | --- | --- | --- |
|  | Baseline | Post | Follow-Up |  | Baseline | Post | |
| Primary Outcome* |  |  |  |  |  |  | |
| Child Impact (Uncertainty) | - | 3.48 (0.94) | 3.05 (0.91) |  | - | 4.99 (0.73) | |
| Family Impact (Uncertainty) | - | 3.34 (0.89) | 3.07 (1.18) |  | - | 5.08 (0.77) | |
| Secondary child outcomes |  |  |  |  |  |  | |
| RULES | 54.41 (11.18) | 49.22 (14.27) | 43.90 (11.26) |  | 60.27 (10.44) | 58.33 (8.10) | |
| IUS-C | 73.93 (18.88) | 69.30 (20.74) | 62.00 (16.57) |  | 78.17 (19.47) | 78.45 (19.69) | |
| ASC-ASD-P total | 50.62 (11.08) | 48.00 (11.82) | 43.05 (8.72) |  | 55.23 (12.22) | 52.35 (12.32) | |
| ASC-ASD-P uncertainty | 20.76 (5.03) | 19.87 (5.45) | 17.48 (4.90) |  | 21.20 (4.60) | 20.70 (4.70) | |
| Secondary parent outcomes |  |  |  |  |  |  | |
| PSOC self-efficacy | 19.83 (3.91) | 22.13 (3.73) | 21.55 (4.11) |  | 17.72 (4.79) | 19.90 (3.40) | |
| PSOC satisfaction | 18.34 (4.84) | 21.04 (5.54) | 21.95 (5.07) |  | 16.52 (5.75) | 15.85 (5.33) | |
| PSOC interest | 14.14 (2.59) | 13.87 (2.30) | 14.69 (2.67) |  | 14.69 (2.67) | 14.40 (3.10) | |
| DASS depression | 3.14 (3.09) | 2.82 (2.54) | 3.20 (3.87) |  | 5.90 (4.13) | 5.53 (4.56) | |
| DASS anxiety | 2.21 (3.27) | 1.82 (2.68) | 2.30 (3.05) |  | 5.28 (4.25) | 4.90 (4.41) | |
| DASS stress | 7.14 (4.98) | 5.77 (3.53) | 5.30 (3.11) |  | 11.14 (4.48) | 10.15 (5.22) | |
| IUS-12 | 22.97 (7.69) | 22.91 (7.75) | 23.19 (7.20) |  | 33.28 (12.73) | 31.35 (12.50) | |

Note. Values are means with standard deviations in parentheses. * Mean value (averaged across four assessors’ blinded ratings) on the scale that underpins the primary outcome variable; values indicate change from baseline to post (Post), and baseline to follow-up (Follow-Up) shown. RULES, Response to Uncertainty and Low Environmental Structure; IUS-C, Intolerance of Uncertainty Scale-Children; ASC-ASD, Anxiety Scale for Children-ASD; PSOC, Parenting Sense of Competence; DASS, Depression, Anxiety and Stress Scale; IUS-12, Intolerance of Uncertainty Scale-12.

Table S3. Parental weekly satisfaction ratings (intervention group only).

| Feedback Question | Sessions | | | | | | | |
| --- | --- | --- | --- | --- | --- | --- | --- | --- |
|  | 1  (*n*=25) | 2  (*n*=23) | 3  (*n*=22) | 4  (*n*=20) | 5  (*n*=18) | 6  (*n*=19) | 7  (*n*=18) | 8  (*n*=19) |
| I liked the content of this session | 5.12 (1.56) | 5.13 (1.18) | 5.59 (0.66) | 5.70 (0.47) | 5.50 (0.62) | 5.68 (0.48) | 5.44 (0.62) | 5.79 (0.41) |
| This session was helpful for my family/child | 5.00 (1.55) | 5.09 (1.24) | 5.59 (0.66) | 5.60 (0.60) | 5.57 (0.59) | 568 (0.48) | 5.44 (0.62) | 5.68 (0.48) |

Note. Likert 6-point scale; 1 indicates ‘strongly disagree’ to 6 indicating ‘strongly agree’. Values indicate mean (SD).

Table S4. Example target uncertain situations

| *Type of Situation* | *Description of Situation* | *Child Impact* | | *Family Impact* | |
| --- | --- | --- | --- | --- | --- |
| School | Performing at school; competitive school sporting events | Wants to participate at school events or competitions, but unable to due to worry about not knowing what the outcome will be. Distress and worry in anticipation of, and during, event; causes sleep challenges. | Significant worry and exhaustion reported by parent; worry over reduced time spent with other children; increasing worry about social isolation from other adults. | |  |
| Sensory | Unfamiliar or public toilets | Unable to use public bathrooms, or bathrooms in unfamiliar homes or places. Can result in refusal to use toilet, even when needed, resulting in accidents. Needs to use a nappy when in unknown public spaces. | Family plans extensively when going out, tending to avoid most public spaces (e.g. shopping centres) and limiting activities the family engages in. Mum worries about child hearing other’s comments on use of nappies (when child is attending school). | |  |
| Social | Attending birthday parties or family gatherings | Asks many questions in advance; may become more irritable as event approaches. Extensive preparation required to support attending a social event, and experiences distress if something not as anticipated. | Family reports feeling exhausted at extent of preparation required. Worry about child’s future and ability to cope without significant support and preparation to attend social events. | |  |
| New Environment | Dining in at cafes or restaurants; going on holidays. | Unable to dine out or go away on holidays. Misses out on trying foods or experiences that parent knows that they would like to try or experience. | Reduced ability to go out as a family; family reports some frustration in limitations in what they can do as a family, and the resultant limitations on siblings’ participation. | |  |

*Note*. Example situations aggregated across multiple participants.
